# Polymer Nanofibers Exhibiting Remarkable Activity in Driving the Living Polymerization under Visible Light and Reusability

**DOI:** 10.1002/advs.201902451

**Published:** 2020-01-27

**Authors:** Lei Xia, Bo‐Fei Cheng, Tian‐You Zeng, Xuan Nie, Guang Chen, Ze Zhang, Wen‐Jian Zhang, Chun‐Yan Hong, Ye‐Zi You

**Affiliations:** ^1^ Hefei National Laboratory for Physical Sciences at the Microscale CAS Key Laboratory of Soft Matter Chemistry Department of Polymer Science and Engineering University of Science and Technology of China Hefei 230026 P. R. China

**Keywords:** nanofibers, photoinduced electron transfer‐reversible addition‐fragmentation chain transfer (PET‐RAFT), photocatalysis, reusability, visible light

## Abstract

Visible light‐driving syntheses have emerged as a powerful tool for organic synthesis and for the preparation of macromolecules under mild and environmentally benign conditions. However, precious but nonreusable photosensitizers or photocatalysts are often required to activate the reaction, limiting its practicality. Here, it is reported that poly(1,4‐diphenylbutadiyne) (PDPB) nanofibers exhibit remarkable activity in driving the living free radical polymerization under visible light. Moreover, PDPB nanofibers are very stable under irradiation of visible light and can be reused without appreciable loss of activity even after repeated cycling. The nanofiber will be a promising photocatalyst with excellent reusability and stability for the reactions driven by visible light.

## Graphical abstract

Polymer nanofibers not only exhibit remarkable activity in driving living free radical polymerization under visible light, but also are very stable under irradiation of visible light and can be reused without appreciable loss of activity even after repeated cycling.

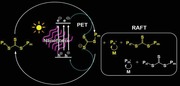

Visible light is a unique natural resource, which is an inexpensive, nonpolluting, abundant, and endlessly renewable source of clean energy. Visible light can drive chemical reaction in distinct activation modes, which are complementary to those traditionally used in the field of transition metal catalysis, thereby enabling reaction development through entirely new mechanistic paradigms.^1^ Under visible light irradiation, many organic molecules and macromolecules can be prepared easily via photochemical paths, which access high energy intermediates that cannot be generated thermally, thereby overcoming large activation barriers in a short period of time, and allowing reactions otherwise inaccessible by thermal processes.^2^ And so, many chemists have begun to use light to drive chemical reactions for the syntheses of small molecules^1^, ^3^ and macromolecules.^4^ However, in most cases, the organic reaction and polymerization systems could not absorb visible light; therefore, photocatalysts or photosensitizers are required to absorb visible light for activating the reaction. MacMillan and co‐workers,[qv: 3a,5] Stephenson and co‐workers,[qv: 3b,c,6] and Yoon and co‐workers,[qv: 3g–i,7] have successfully designed several transition metal complexes, which have shown excellent performance in synthesis of small molecules driven by visible light. For polymer synthesis, visible light‐driven polymerization not only synthesizes polymers with predictable molecular weight, and well‐defined end‐group functionality, but also can spatially and temporally control the macromolecular synthesis. Hawker and co‐workers,^8^ Fors and co‐workers,^9^ Matyjaszewski and co‐workers,^10^ Yagci and co‐workers,^11^ Miyake and co‐workers,^12^ Boyer and co‐workers,^13^ Qiao and co‐workers^14^ and many others^15^ have very successfully developed efficient photopolymerization system under visible light. Basically, it is preferable to develop metal‐free and reusable photocatalytic systems to lower the cost and prevent metal contamination in the polymer products.[qv: 11j,15e,16] However, most of developed catalysts are not reusable.[qv: 4c,d,17] Conducting polymer nanofibers can be cheaply and conveniently prepared in large scale via chemical or electrochemical approaches, also they have high conductivities; excellent thermal, photo, and chemical stability; flexibility; elevated carrier mobility; biocompatibility; as well as unique electrochemical and optical properties.^18^ Some conducting polymers were found to be very efficient in the degradation of various textile dyes under irradiation, and some conducting poly(*p*‐phenylene)s can drive pinacol coupling benzaldehyde under visible light.^19^ Therefore, we can envision that these unique properties of conducting polymers could render them to be very promising in catalyzing the photopolymerization reaction under visible light.

Boyer and co‐workers have invented the photoinduced electron transfer‐reversible addition‐fragmentation chain transfer (PET‐RAFT)[qv: 13g] polymerization using transition metal complexes[qv: 13d,g,20] or organic dyes[qv: 13c,e,h] as photocatalyst, including Ir(ppy)_3_, Ru(bpy)_3_^2+^, ZnTPP, eosin Y, and chlorophyll with reduction potential of −1.72, −0.81, −1.1, −1.32, and −1.1 V versus saturated calomel electrode (SCE, in acetonitrile), respectively. The reduction potential values of most thiocarbonylthio compounds are among −0.4 to −0.9 V versus SCE (in acetonitrile). Generally, *S*,*S*′‐bis(α,α′‐dimethyl‐α″‐acetic acid)‐trithiocarbonate (BDMAT) has reduction potential value of −0.88 V versus Ag/AgCl (in acetonitrile) (Figure S5, Supporting Information). We prepared poly(1,4‐diphenylbutadiyne) nanofibers (PDPB‐NF) in nanoreactors of mesophases as shown in **Figure** **1**A,B,^21^ and its reduction potential values can be easily tuned by polymerization degree.[qv: 21b] For example, we prepared PDPB‐NFs with 4, 6 polymerization degree, which have reduction potential values of −1.23 V versus Ag/AgCl (in acetonitrile) (Figure S6, Supporting Information) and 1.16 V versus Ag/AgCl (in acetonitrile) (Figure S7, Supporting Information) as shown in Figure 1E. Under visible light irradiation, PDPB‐NF6 (PDPB‐NF with 6 polymerization degree) could generate conduction‐band electrons (e^−^) and valence‐band holes (h^+^) upon excitation. Because the reduction potential values of the excited state PDPB‐NF6 (E(PDPB‐NF6*/(PDPB‐NF6)^•+^) = −0.98 V versus Ag/AgCl, in acetonitrile) are lower than that of BDMAT, in the presence of BDMAT and monomers, based on PET‐RAFT mechanism, BDMAT could accept an electron from conduction‐band electrons (e^−^), producing a radical to initiate RAFT polymerization, in which thiocarbonylthio compound acts as both initiator and chain transfer agent. Simultaneously, the radical could also be deactivated by holes to regenerate the thiocarbonylthio compound and ground‐state PDPB‐NF6, and the whole catalytic cycle would restart again (**Scheme** **1**).

**Figure 1:**
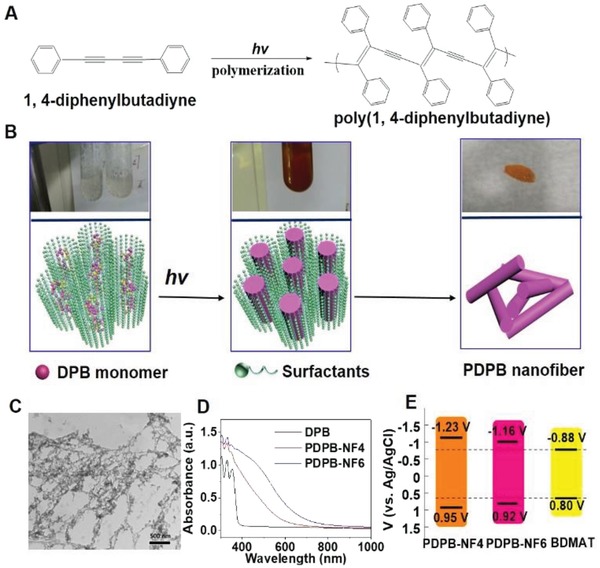
A) Schematic of polymerization of diphenylbutadiyne (DPB). B) Schematic of synthesis of PDPB nanofibers in soft template. C) TEM photograph of PDPB‐NF6 nanofibers. D) UV–vis diffuse reflectance spectra of DPB, PDPB‐NF4, and PDPB‐NF6. E) HOMO and LUMO band position of PDPB‐NF4, PDPB‐NF6, and BDMAT.

**Scheme 1:**
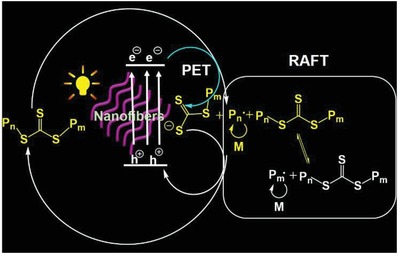
Schematic of poly(1,4‐diphenylbutadiyne) nanofibers (PDPB‐NF) driving photo‐induced electron transfer reversible addition‐fragmentation chain transfer (PET‐RAFT) polymerization.

UV–vis diffuse reflectance spectroscopy (Figure 1D) was used to investigate the absorption performance of the formed PDPB‐NFs. With the increase of degree of polymerization, the absorption region was extended from ultraviolet light region to visible light region. It is clear that PDPB‐NF6 has strong absorption in the range of 400–800 nm. Due to the low band gap, excess electrons and holes formed in the PDPB‐NF6 under irradiation by visible light. In order to identify whether there is electron transfer between PDPB‐NF6 and BDMAT, the fluorescence quenching study was carried out. Generally, the fluorescence signal^22^ of conducting polymer comes from the recombination of photogenerated electrons and holes, and the fluorescence peak does not change under irradiation by different excitation wavelength (Figure S12, Supporting Information). But in these experiments, we have found that the intensity of the fluorescence emission peak was synchronously decreased (Figure S13, Supporting Information) with the increase in the concentration of the BDMAT, indicating that there was electron transfer between PDPB‐NF6 and BDMAT. Compared with PDPB‐NF4, PDPB‐NF6 is more efficient as shown in Table S1, Supporting Information. Because of lower absorption in visible light region, PDPB‐NF4 has lower utilization efficiency, which results in poor activity. Compared with PDPB6 (PDPB with 6 polymerization degree synthesized by bulk polymerization), PDPB‐NF6 possess a 1D nanofiber structure; the width of the nanofiber is ≈24 nm while PDPB6 do not have nanostructure. The nanofiber structure could make charge transfer much faster due to the shorter carrier diffusion distance probably in nanostructures.^23^ On the other hand, PDPB‐NF6 with 1D nanofiber structure exhibited a higher activity than PDPB6, which also might be due to the larger size and the presence of more defects in PDPB6 favoring higher e^−^–h^+^ recombination. This dependence of the photocatalytic activity on the size and morphology of the structure has also been observed in the case of semiconductors such as TiO_2_.^24^ To verify the above hypothesis, methyl viologen (MVCl_2_), a fast electron acceptor and quencher, for checking the electron transfer between Ru complexes and amine,^25^ was used to measure the value of quenching rate constant, which is related with the capability of electron transfer.^23^ The results (Figure S14, Supporting Information) showed that the plot of the ratio *I*_0_/*I* versus the MVCl_2_ concentration was linear, the slope of fitting curve is indicative of *k*_q_τ^0^. The greater the slope, the higher the efficiency of electron transfer. The slope of PDPB‐NF6 (0.283) is much higher than that of PDPB‐NF4 (0.216) and PDPB6 (0.187), and hence PDPB‐NF6 has fast charge transfer efficiency and high activity. Furthermore, when a very small amount of methyl viologen was added into polymerization mixture in the presence of PDPB‐NF6, the rate of polymerization dramatically decreased (Table S2, Supporting Information), indicating that electrons did not transfer to BDMAT, but transferred to MV^2+^, forming MV^•+^, which could not act as an initiate species.

In order to investigate the activity of PDPB‐NF driving PET‐RAFT polymerization under visible light, we chose *N*,*N*‐dimethylacrylamide (DMA) as model monomer and water as solvent. First, DMA and BDMAT fully dissolved in water without any PDPB‐NF were irradiated by visible light using a 100–300 W lamp with light intensity of 100 W at a fixed distance of 25 cm between lamp and reactor at room temperature; almost no polymer was obtained because of very low monomer conversion (3.2%) (**Table** **1**, Entry 1). However, when mixture of DMA and BDMAT in water was irradiated by visible light in the presence of PDPB‐NF6, polymerization did occur, and PDMA with molecular weight of 5300 g mol^–1^ and narrow molecular distribution (*Ð* = 1.14) were obtained in 2.0 h (Table 1, Entry 2; Figure S9, Supporting Information). The monomer conversion and molecular weight increased with the increase of irradiation time. In addition, 2‐hydroxyethyl acrylate (HEA) and *N*‐(3‐(dimethylamino)propyl)acrylamide (DPAA) were used in this polymerization system as well (Table 1, Entry 4 and 5; Figure S10 and S11, Supporting Information), and molecular weights were well controlled and *Ð*s were narrow. As shown in **Figure** **2**A, a typical first‐order kinetic trace was observed according to the plot of ln([*M*]_0_/[*M*]_t_) as a function of reaction time (the apparent propagation rate *k*_p(visible light)_ = 0.640 h). GPC curves shifted to high molecular weights along with the extension of polymerization time. The dispersity (*Đ*) is below 1.30. The evolution of experimental molecular weights to monomer conversion showed a linear relationship as well (Figure 2B), which was in accord with the behaviors of controlled/living polymerization. Additionally, polymerization can be also carried out smoothly under green light irradiation (Figure S16, Supporting Information) with lower apparent propagation rate (*k*_p(green light)_ = 0.158 h). All these results have exhibited that PDPB‐NF6 can drive the PET‐RAFT polymerization under visible light. Commonly, most of organic photocatalysts are not very stable under light irradiation, which can degrade easily. Under the irradiation of visible light, 45% of pure eosin Y was degraded after 2.0 h (Figure S15, Supporting Information), but its stability can be improved by being loaded in nanoparticles[qv: 16e] or adding stabilizer.[qv: 13j] However, almost no obvious photo‐degradation of PDPB‐NF6 was observed after 2.0 h, indicating that PDPB‐NF6 was very stable under the irradiation of visible light. To further investigate the end‐group fidelity, chain extension of PDMA polymer was carried out using HEA as second monomer. It is very clear that the molecular weight trace shifted to the higher molecular weight side (Figure 2D), which indicated remarkably well‐controlled chain extension due to high end‐group fidelity.

**Table 1:** Examples of polymers synthesized by PDPB‐NF6 catalyzed PET‐RAFT polymerization under visible light

Entry^a)^	Monomer	Time [h]	Conv.^b)^ [%]	*M*_n.theo_ ^b)^ [g mol^–1^]	*M*_n.GPC_ (*Đ*)^c)^ [g mol^–1^]
1^d)^	DMA	0.5	3.2	599	–^e)^
2	DMA	0.5	33.1	3559	1050(1.28)
3	DMA	2.0	80.3	8231	5300(1.14)
4	HEA	2.0	64.9	7810	7900(1.10)
5	DPAA	1.0	40.6	6520	3700(1.26)

^a)^ Reaction conditions: [*M*]_0_:[BDMAT]_0_ = 100:1, [*M*]_0_ = 0.5 M, 1.0 mg PDPB‐NF6 dispersed in 1 mL water. Polymerization was carried out at room temperature with visible light irradiation

^b)^ Monomer conversion and *M*_n,theo_ determined by ^1^H NMR spectroscopy; *M*_n,theo_ = [*M*]_0_/[BDMAT]_0_ × *MW*_M_ × Conv + *MW*_BDMAT_, where [*M*]_0_, [BDMAT]_0_, *MW*_M_, Conv, and *MW*_BDMAT_ are the initial monomer concentration, initial BDMAT concentration, molar mass of the monomer, monomer conversion, and molar mass of BDMAT, respectively

^c)^ *M*_n,GPC_ and *Đ* were determined by GPC with PS standards

^d)^ Polymerization was carried out in the absence of PDPB‐NF6

^e)^ Not determined.

**Figure 2:**
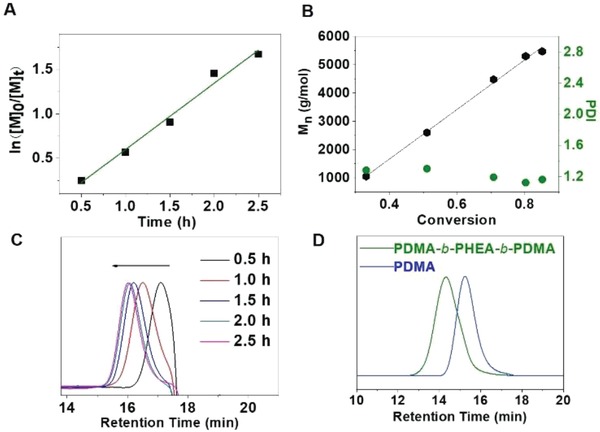
A) The plot of ln([*M*]_0_/[*M*]_t_) as a function of reaction time. B) The relationship of *M*_n,GPC_ and *Đ* versus monomer conversion. C) GPC traces at different times of irradiation. D) GPC traces of before and after chain extension.

The photo‐triggered chemical reactions generally have spatiotemporal effect, and PET‐RAFT polymerization can be easily switched ON and OFF. As shown in **Figure** **3**A, the initiation and termination of the polymerization could be controlled by the light “ON” and “OFF”, respectively. At the same time, final polymer with low *Đ* (*Đ* = 1.21, Figure 3B) was in accord with the feature of controlled/living polymerization.

**Figure 3:**
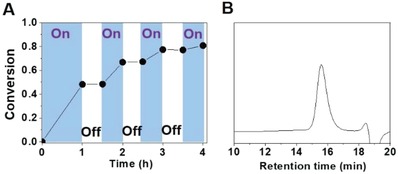
A) Conversion versus time of polymerization in the presence (“On”) or in the absence (“Off”) of visible light. B) GPC curve of final polymer (*M*_n,GPC_ = 5700 g mol^–1^, *Đ* = 1.21).

That the photocatalysts can be efficiently recycled and reused for repeated cycles without appreciable loss of activity is crucial for industrial applications, where stable and good recyclability of photocatalysts is highly desirable. However, in most cases, it is difficult for photocatalysts to be recycled and reused for many repeated cycles. NMR and FTIR spectra of PDPB‐NF6 before and after PET‐RAFT polymerization are shown in **Figure** **4**A,C,D, and it is clear that there were no big differences before and after polymerization. Furthermore, TEM image (Figure 4B) has shown that the morphology of the nanofiber structures of PDPN‐NF6 remained the same after repeated photocatalytic cycling. These results verify that the PDPB nanofibers are very stable photocatalyst. On the other hand, due to the low solubility in water, PDPB‐NF as a heterogeneous catalyst can be easily isolated by centrifugation. And we found that the photocatalytic activity of PDPB‐NF6 did not decrease very much even after several cycles (Figure 4E,F), which indicates that that PDPB nanofibers can be efficiently recycled and reused for repeated cycles without appreciable loss of activity. This feature is crucial for industrial applications, where stability and good recyclability of photocatalysts is highly desirable.

**Figure 4:**
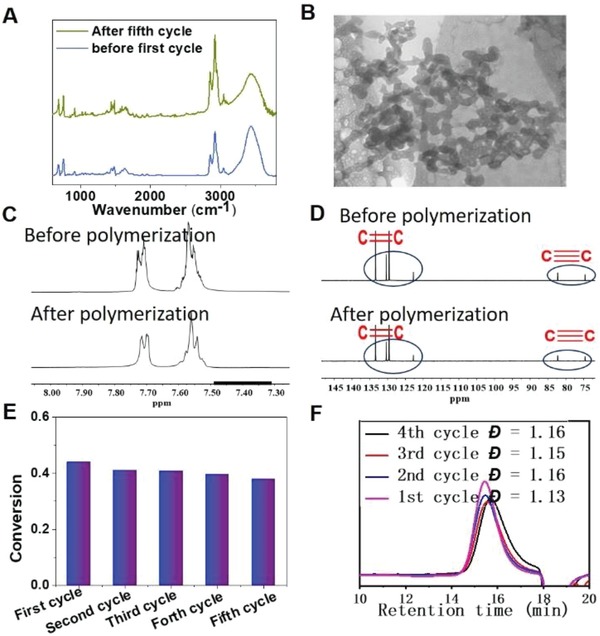
Repeated use of PDPB‐NF6 in PET‐RAFT polymerization of DMA. A) IR spectra of PDPB‐NF6 before first cycle and after fifth cycle. B) TEM image of PDPB‐NF6 after fifth cycle. C) ^1^H NMR and D) ^13^C‐NMR spectra of PDPB‐NF6 before and after polymerization. E) Monomer conversion of each cycle. F) GPC curves of PDMA.

In conclusion, the polymer nanofibers can drive the living/controlled radical polymerization under visible‐light in water without any assistance of sacrificial reagents or precious metal cocatalysts. It has exhibited remarkable activity in catalyzing the living free radical polymerization of common water‐soluble monomers including acrylamides and acrylates. Moreover, this technique showed excellent control over molecular weight and polydispersity (*Đ*) and high group fidelity with successful chain extension. Moreover, these polymer nanofibers are very stable even after repeated cycling and could be reused without appreciable loss of activity. This finding may present a promising strategy toward the development of efficient and reusable photocatalyst for polymerization driven by visible light with great environmental benefits that can directly harvest energy from solar light.

## Conflict of Interest

The authors declare no conflict of interest.

## Supporting information

Supporting Information
