# Supplementary material for: Polymer Nanofibers Exhibiting Remarkable Activity in Driving the Living Polymerization under Visible Light and Reusability
Source: Adv Sci (Weinh). 2020 Jan 27;7(6):1902451. doi: 10.1002/advs.201902451 (PMC7080551; doi:10.1002/advs.201902451)
Supplement: Supplementary file 1 — Supporting Information [file ADVS-7-1902451-s001.pdf]

Copyright WILEY-VCH Verlag GmbH & Co. KGaA, 69469 Weinheim, Germany, 2020.

ADVANCED SCIENCE Open Access

Supporting Information

for *Adv. Sci.,* DOI: 10.1002/advs.201902451

Polymer Nanofibers Exhibiting Remarkable Activity in Driving the Living Polymerization under Visible Light and Reusability

Lei Xia, Bo-Fei Cheng, Tian-You Zeng, Xuan Nie, Guang Chen, Ze Zhang, Wen-Jian Zhang,* Chun-Yan Hong,* and Ye-Zi You**

Copyright WILEY-VCH Verlag GmbH & Co. KGaA, 69469 Weinheim, Germany, 2018.

Supporting Information

Polymer Nanofibers Exhibiting Remarkable Activity in Driving the Living Polymerization under Visible Light and Reusability

Lei Xia, Bo-Fei Cheng, Tian-You Zeng, Xuan Nie, Guang Chen, Ze Zhang, Wen-Jian Zhang*, Chun-Yan Hong*, Ye-Zi You**

Materials

1, 4-Diphenylbutadiyne (DPB) (Aldrich, 99%), benzoin methyl ether (BME) (Admas, 98%), N, N-dimethyl acrylamide (DMA) (Aldrich, 99%), 2-hydroxyethyl acrylate(HEA) (Aldrich, 99%), esion Y (Aldrich, 98%), N-(3-(dimethylamino)propyl)acrylamide (DPAA) (TCI, 98%), N,N-dimethyl-ϒ,ϒ’-dipyridyliumdichloride (methylviologen, $MVCl_2$) (Aldrich, 98%), tetrabutylammonium hexafluorophosphate (Energy, 98%), 4'-azobis(4-cyanovaleric acid) (Adamas, 98%), tetrabutylammonium hydrogen sulfate (Energy, 99%), carbon disulfide (AR), chloroform (AR), acetone (AR), mineral ether (AR), sodium hydroxide (AR), sodium dodecyl sulfate (CP), sodium chloride (AR), cyclohexane (AR), pentanol-1 (AR) were obtained from Shanghai Chemical Reagent Co.

Experimental Procedures

Synthesis of S, S'-bis(α, α'-dimethyl-α"-acetic acid)-trithiocarbonate (BDMAT).

Carbon disulfide (13.77 g, 0.18 mol), chloroform (58.81 g, 0.45 mol), acetone (26.26 g, 0.45 mol), and tetrabutylammonium hydrogen sulfate (1.21 g, 3.6 mmol) were mixed with 12 mL of mineral ether in a 500 mL round bottom flask. NaOH solution (50%, 100.8 g) was added into the mixture for 1.0 h while maintaining the temperature at 25 ^{o}C. Then, the reaction was maintained at 22-25 ^{o}C for 10 h while being stirred with a magnetic stirrer. Water (90 mL) was added to dissolve the yellow solids, followed by adding hydrochloric acid (12 mL) to acidify the aqueous solution and yield crystalline solids. After filtration and washing three times by water, the crude compound was purified by recrystallization three times in mixture of acetone and mineral ether (4:1 v/v). Yield: 53%. The ^{1}H NMR result provided in Figure S1.

Synthesis of PDPB-NF4, PDPB-NF6 and PDPB6[21a]

Sodium dodecyl sulphate (1.0 g) was dissolved in 2 mL of 0.3 M NaCl aqueous solution in a quartz tube. After slow agitation at 65 ^{o}C until the surfactant has completely dissolved to give a transparent and viscous micellar solution. The subsequent addition of cyclohexane containing monomer (1, 4-diphenylbutadiyne (DPB) (10% of mass)) and initiator benzoin methyl ether (BME) (5% of mass) in the micellar solution under stirring leads to a white unstable emulsion at 1000 rpm. A co-surfactant, pentanol-1 (300 μL), was then added to the mixture, which was then strongly vortexed for a few minutes. This led to a perfectly colourless, translucent, birefringent and stable gel: a hexagonal mesophase. The doped mesophases with the monomer and the initiator for polymerization were used as soft templates to synthesize polymer nanostructures induced by irradiation using an OSRAM ULTRA-VITALUX (100-300 W) lamp at a distance of 20 cm for one week for PDPB-NF4

and two weeks for PDPB-NF6. After reaction, the materials were extracted in water-ethanol mixture, centrifuged, and then washed several times to eliminate the surfactant, co-surfactant and salt. Similar photopolymerization route just without soft templates was carried out to synthetize PDPB powder (PDPB6).

General procedures for photo-induced living polymerization catalyzed by various catalysts.

The polymerization ware conducted in a glass sealed tube, charged with water (3 mL), monomer (DMA, HEA, DPAA,) (1.5 mmol), BDMAT (4.23 mg, 0.015 mmol), and catalyst (3.0 mg). The mixture was deoxygenated by three freeze-pump-thaw cycles and sealed under vacuum. The mixture was then irradiated by OSRAM ULTRA-VITALUX (100-300 W) lamp at room temperature. After stop polymerization, catalyst was removed by centrifugation. Polymers were obtained by precipitation and analyzed by ^{1}H NMR and GPC.

Redox potentials of BDMAT and various catalysts measured by cyclic voltammetry.

Samples (1 mg/mL in ethanol) were drop-casted onto the GC-electrode and then dried. The GC-electrode was immersed into the electrochemical cell containing acetonitrile with 0.1 M tetrabutylammonium hexafluorophosphate ($TBAPF_6$). The compounds were electrochemically reduced prior to being oxidized between −2.0 V and +2.0 V at a scan rate of 100 mV/s.

General procedures for photo-induced living polymerization catalyzed by PDPB-NF6 in presence of fast electron acceptor, methylviologen ($MVCl_2$).

The polymerization was conducted in a sealed glass tube, charged with water (3 mL), DMA (148.5 mg, 15.0 mmol), BDMAT (4.23 mg, 0.015 mmol), $MVCl_2$ (2.01 μg, 0.078 μmol), and PDPB-NF6 (3.0 mg). The mixture was deoxygenated by three freeze-pump-thaw cycles and

sealed under vacuum. The mixture was then irradiated by OSRAM ULTRA-VITALUX (100-300 W) at room temperature. After stop polymerization, catalyst was removed by centrifugation. Polymers were obtained by precipitation and analyzed by ^{1}H NMR and GPC.

Investigation of electron transfer mechanism using fluorescence quenching study.

A series of plastic tube containing PDPB-NF6 (at the same concentration of 0.125 mg/mL in EtOH) and quencher, $MVCl_2$ (varying concentrations from 0 to 3.89 mM). The mixture was degassed by N_2 for 20 min, then fluorescence properties were measured by fluorescence spectrometer.

General procedures for kinetic study of photo-induced living polymerization catalyzed by PDPB-NF6.

The polymerization were conducted in a glass sealed tube, charged with water (3 mL), DMA (148.5 mg, 15.0 mmol), BDMAT (4.23 mg, 0.015 mmol), and PDPB-NF6 (3.0 mg). The mixture was deoxygenated by three freeze-pump-thaw cycles and sealed under vacuum. The mixture was then irradiated by OSRAM ULTRA-VITALUX (100-300 W), or green light emitted by this lamp through a filter at room temperature. Aliquots were withdrawn by nitrogen-purged syringes from the reaction mixture at predetermined interval times, Polymers were obtained by precipitation and analyzed by ^{1}H NMR, GPC.

General procedures for chain extension of PDMA via photo-induced living polymerization catalyzed by PDPB-NF6.

A typical experiment procedures for synthesizing the triblock copolymer PDMA-*b*-PHEA-*b*-PDMA as follow: HEA (417.6 mg, 3.6 mmol), PDMA (macro-RAFT agent, M_n = 6000 g/mol, $Đ$ = 1.12, 54 mg, 0.009 mmol), PDPB-NF6 (1.0 mg) and water (1 mL) were added into a tube. Subsequently, the mixture was degassed by three freeze-pump-thaw cycles. The tube was

sealed under vacuum and irradiated by OSRAM ULTRA-VITALUX (100-300 W) at room temperature. After stop polymerization, catalyst was removed by centrifugation. The resulting product was obtained by precipitation and analyzed by ^{1}H NMR and GPC.

General procedures for recycle utilization of PDPB-NF6 in photo-catalyzing living radical polymerization.

The polymerization were conducted in a glass sealed tube, charged with water (3 mL), DMA (297 mg, 3.0 mmol), BDMAT (2.1 mg, 0.0075 mmol), and PDPB-NF6 (1 mg/mL). The mixture was deoxygenated by three freeze-pump-thaw cycles and sealed under vacuum. The mixture was then irradiated by OSRAM ULTRA-VITALUX (100-300 W) at room temperature. After stop polymerization, catalyst was removed by centrifugation, then dried and used again. Polymers were obtained by precipitation and analyzed by ^{1}H NMR and GPC.

Photobleaching studies of eosin Y and catalysts.

The photobleaching experiments were conducted in a glass sealed tube, charged with eosin Y (1×10^{-5} M) and catalysts (1 mg/mL) in DMF, respectively. The mixture was deoxygenated by three freeze-pump-thaw cycles and sealed under vacuum. The mixture was then irradiated by OSRAM ULTRA-VITALUX (100-300 W) at room temperature. Aliquots were withdrawn by syringe from the reaction mixture at predetermined interval times, and analyzed by UV-vis Spectroscopy.

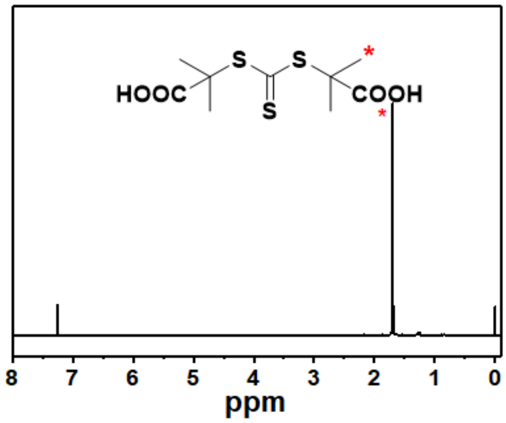

Figure S1. ^{1}H NMR spectrum of BDMAT.

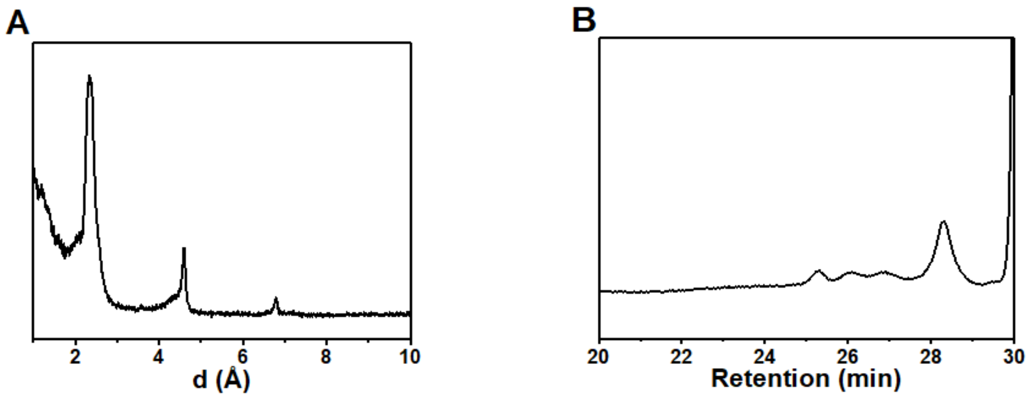

Figure S2. (A) SXRD pattern of hexagonal phases that be used to synthesis of PDPB-NF4. (B) GPC curve of PDPB-NF4 ($M_{n,GPC}$ = 750 g/mol, DP = 4).

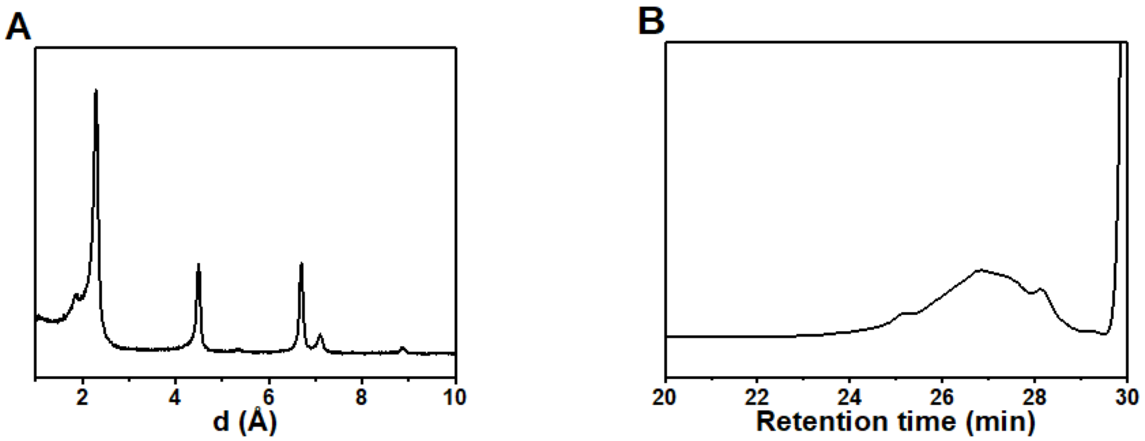

Figure S3. (A) SXRD spectrum of gel that be used to synthesis of PDPB-NF4. (B) GPC curve of PDPB-NF6 ($M_{n,GPC}$ = 1100 g/mol, DP = 6).

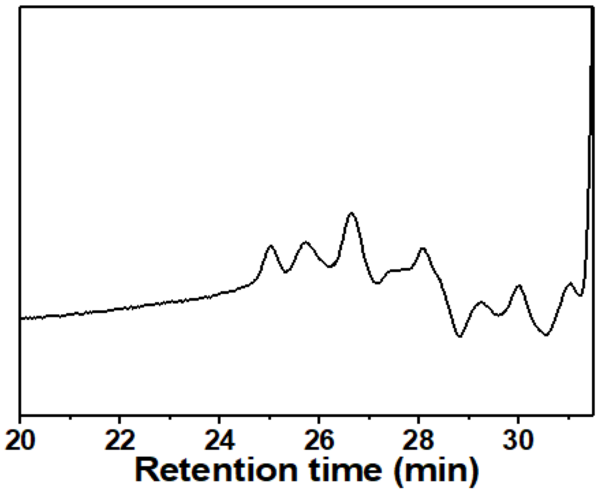

Figure S4. GPC curve of PDPB6 ($M_{n,GPC}$ = 1210 g/mol, DP = 6).

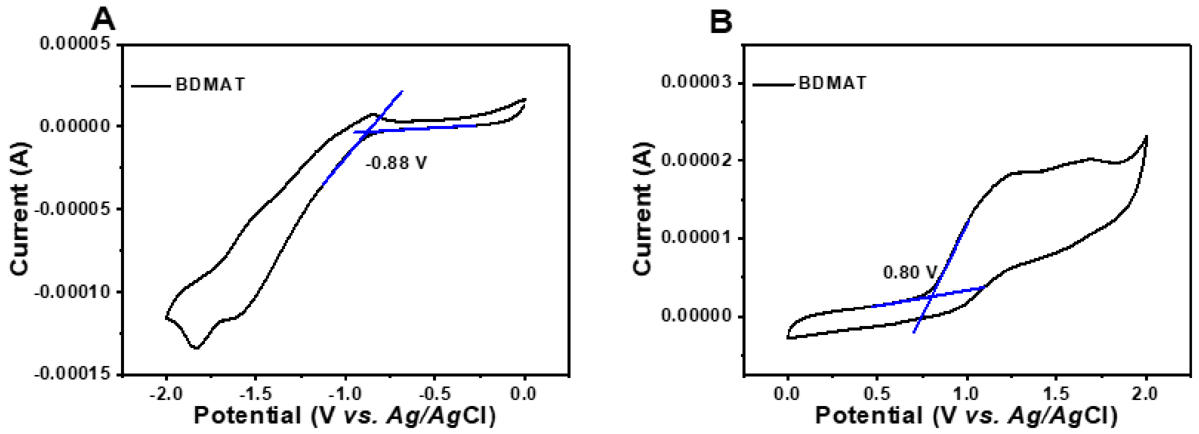

Figure S5. Cyclicvoltammograms of BDMAT.

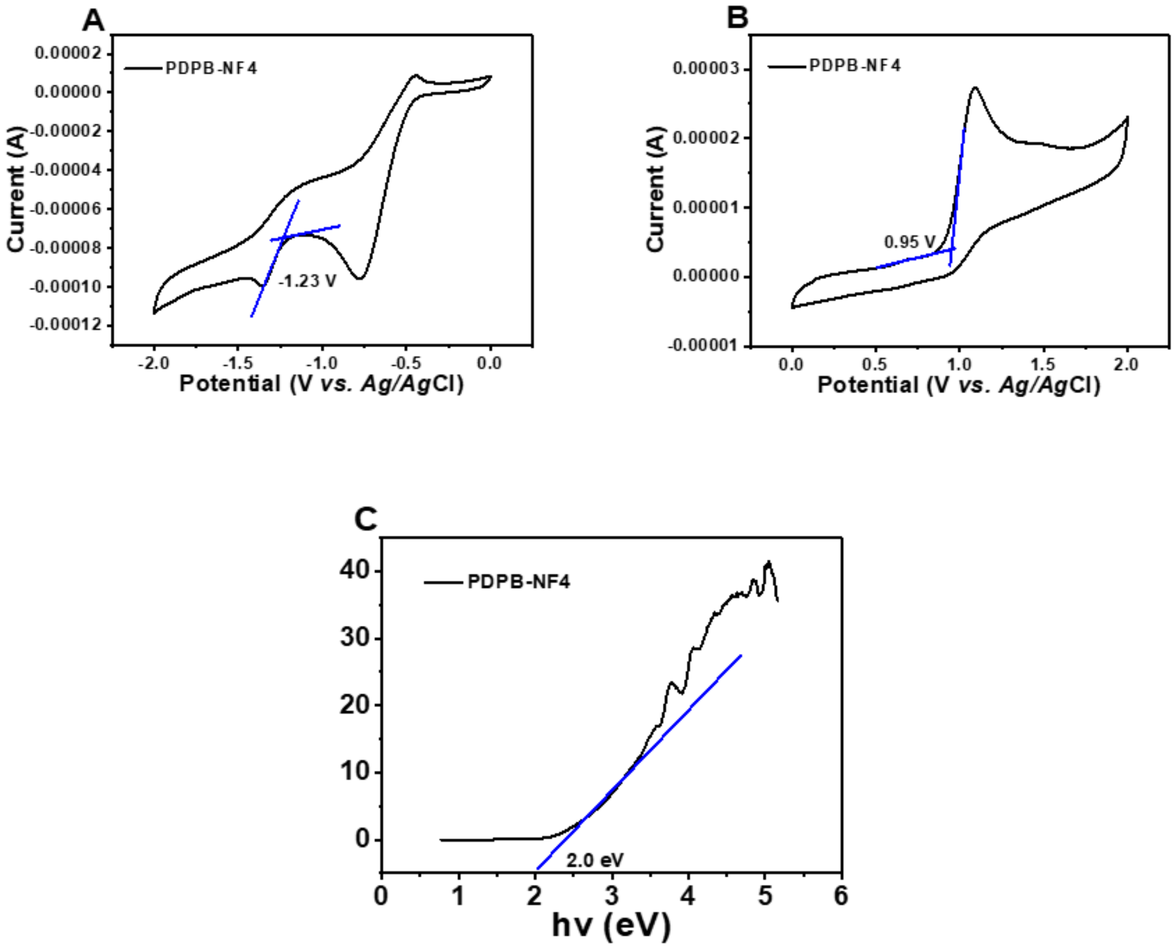

Figure S6. Cyclicvoltammograms and E_g value of PDPB-NF4. E(PDPB-NF4*/(PDPB-NF4)$^{\bullet+}$) = –0.98 V vs Ag/AgCl (in acetonitrile).

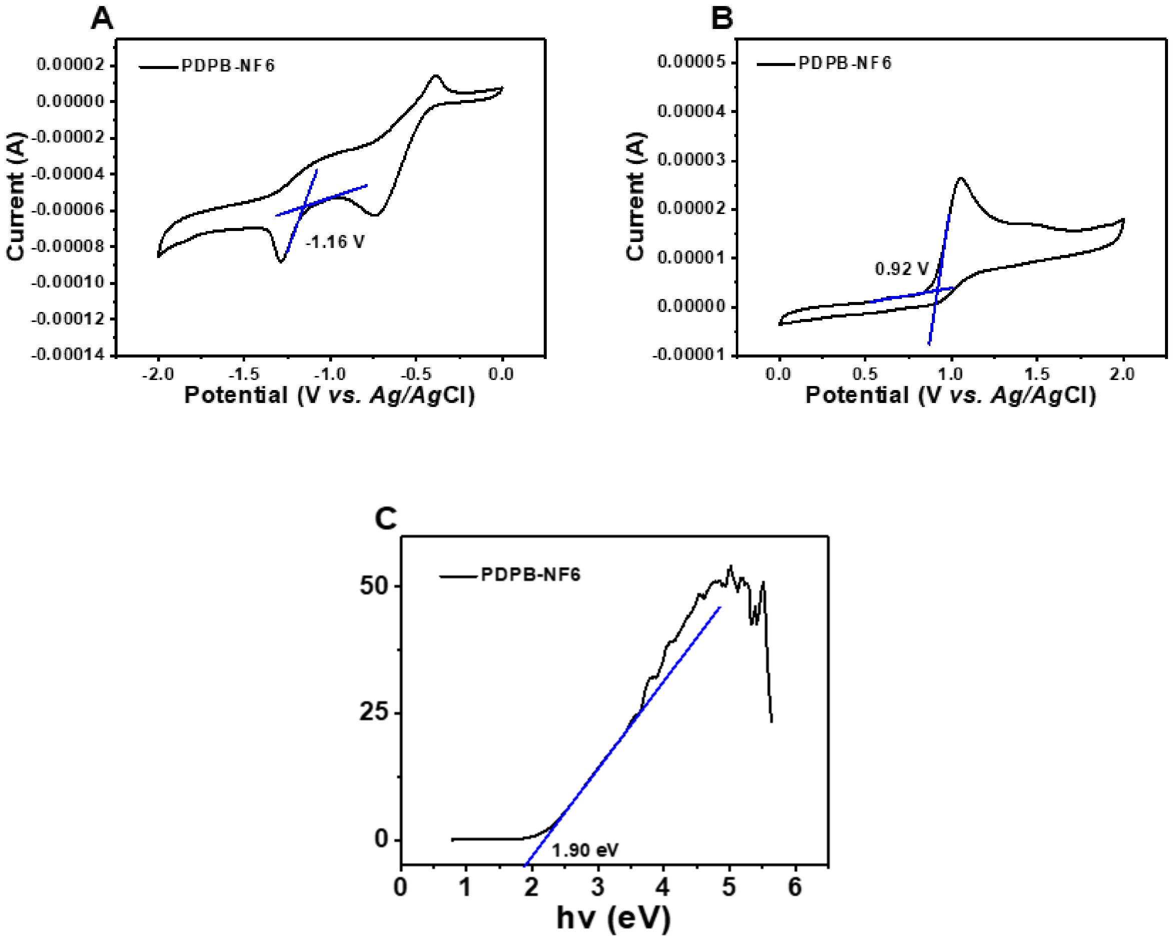

Figure S7. Cyclicvoltammograms of E_g value PDPB-NF6. E(PDPB-NF6*/(PDPB-NF6)$^{\bullet+}$) = –0.98 V vs Ag/AgCl (in acetonitrile)

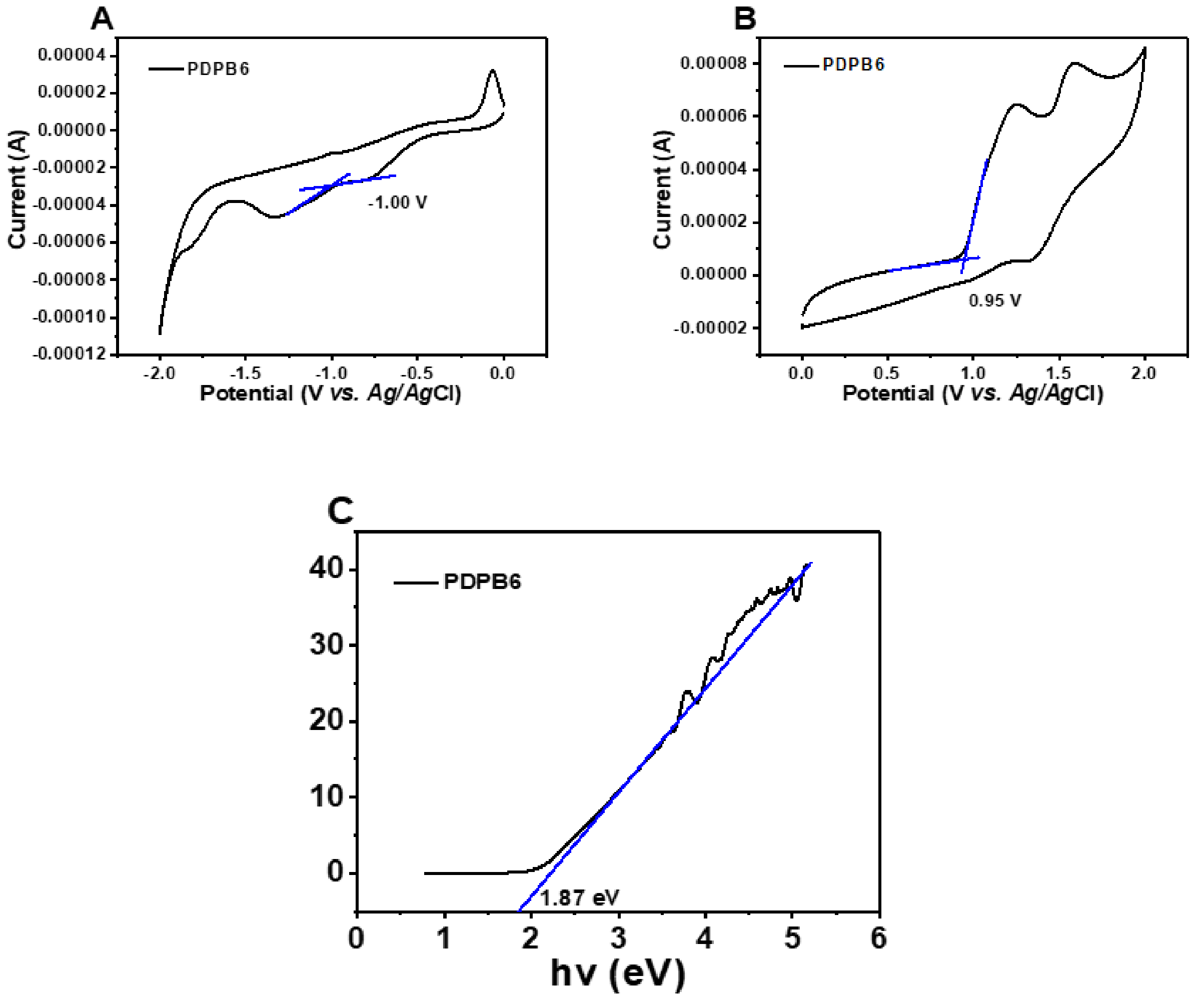

Figure S8. Cyclicvoltammograms of E_g value PDPB6. E(PDPB6*/(PDPB6$^{\bullet+}$) = –0.92 V vs Ag/AgCl (in acetonitrile).

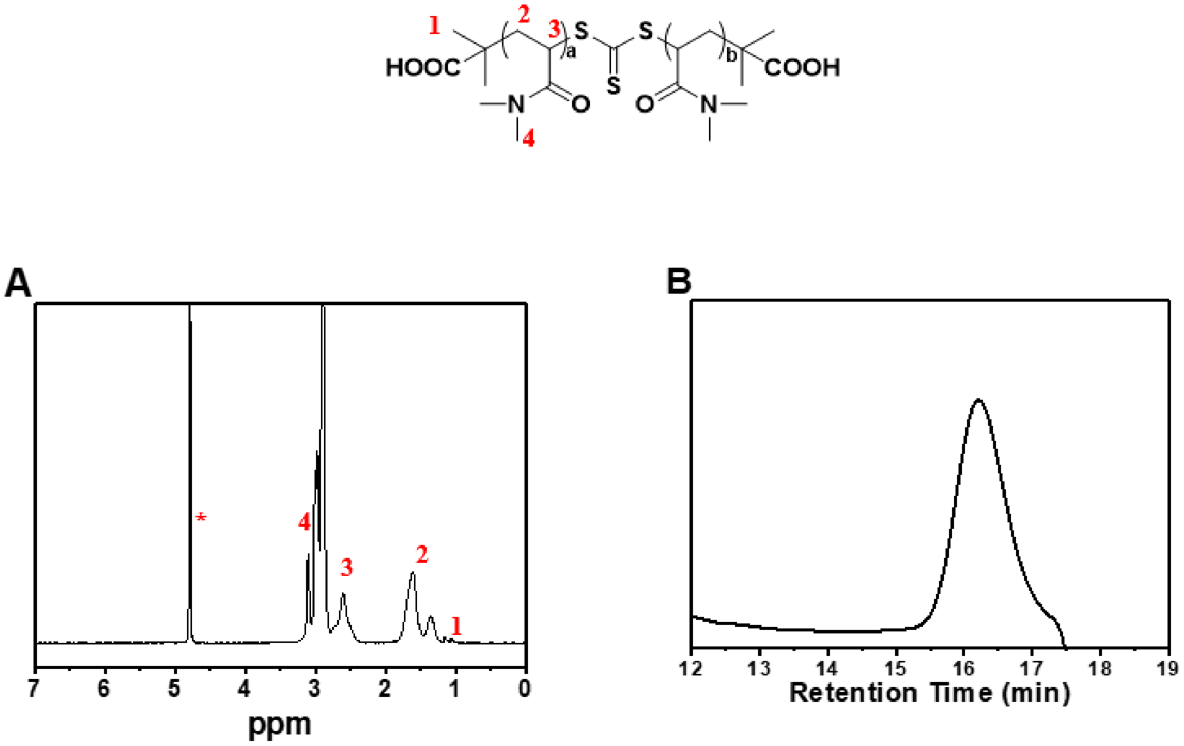

Figure S9. (A) ^{1}H NMR spectrum and (B) GPC curve of PDMA ($M_{n,GPC}$ = 5300 g/mol, $Đ$ = 1.14).

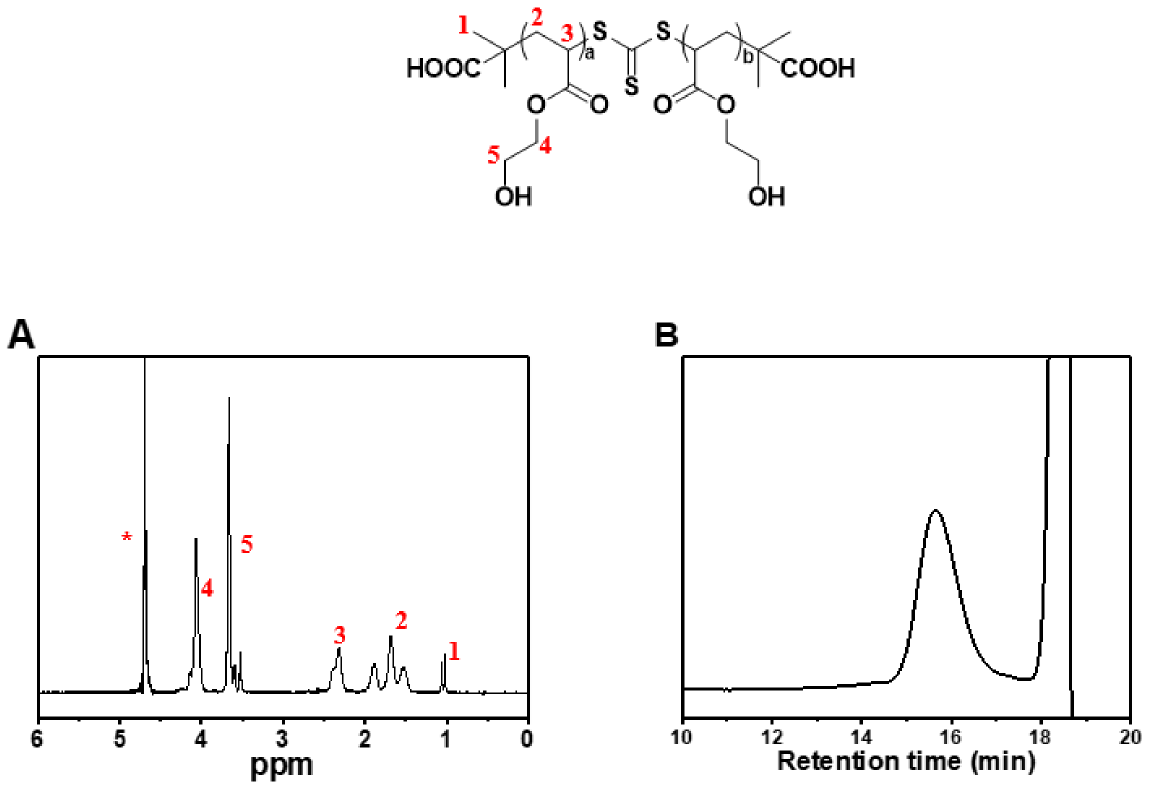

Figure S10. (A) ^{1}H NMR spectrum and (B) GPC curve of PHEA ($M_{n,GPC}$ = 7900 g/mol, $Đ$ = 1.10).

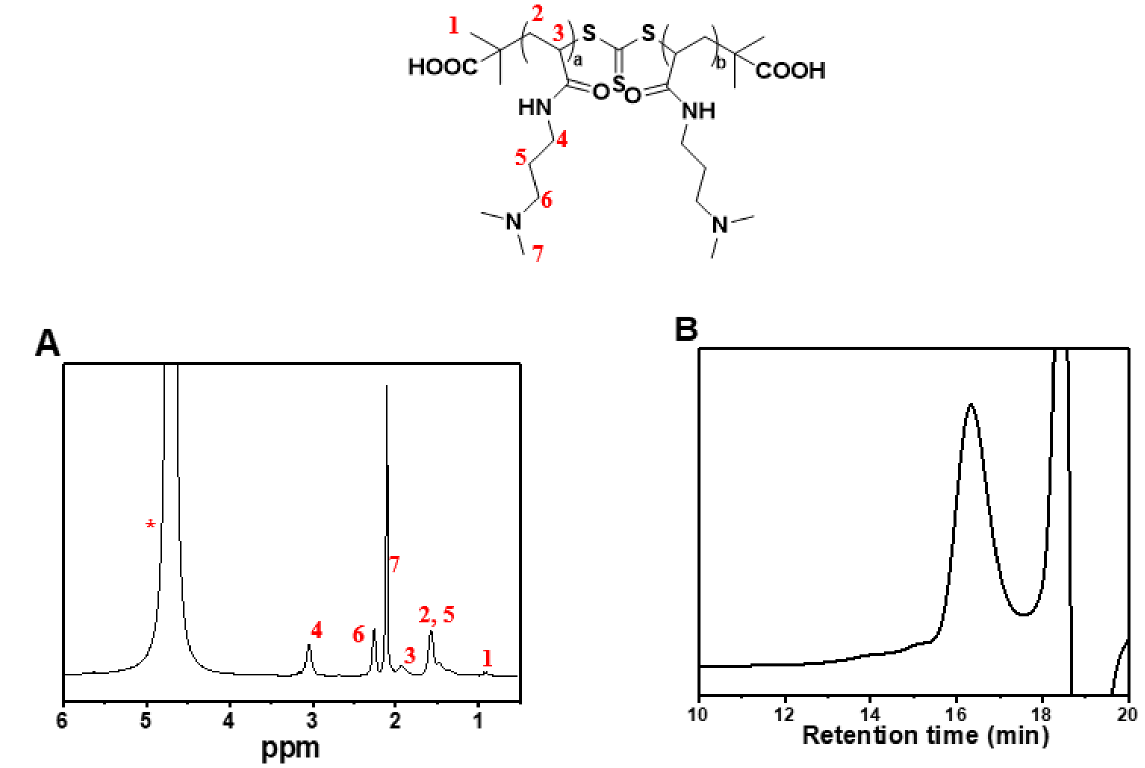

Figure S11. (A) ^{1}H NMR spectrum and (B) GPC curve of PDPAA ($M_{n,GPC}$ = 3700 g/mol, $Đ$ = 1.26).

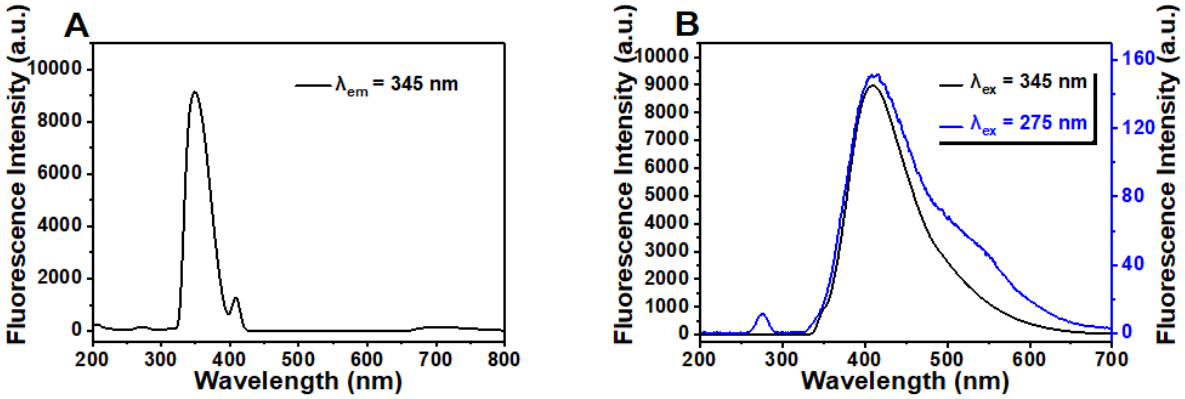

Figure S12. (A) Excitation and (B) emission spectra of PDPB-NF6 in EtOH.

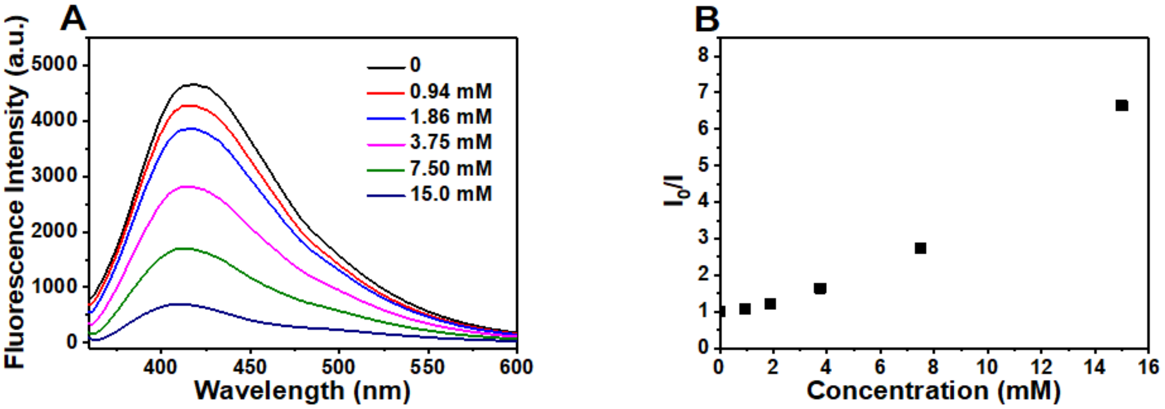

Figure S13. (A) Fluorescence quenching study of PDPB-NF6 (0.25 mg/mL) in EtOH with varying concentrations of BDMAT. (B) Plots of the ratio I_0/I versus quencher concentration. I_0 and I correspond to the emission intensity of PDPB-NF6 in the absence and presence of BDMAT, respectively.

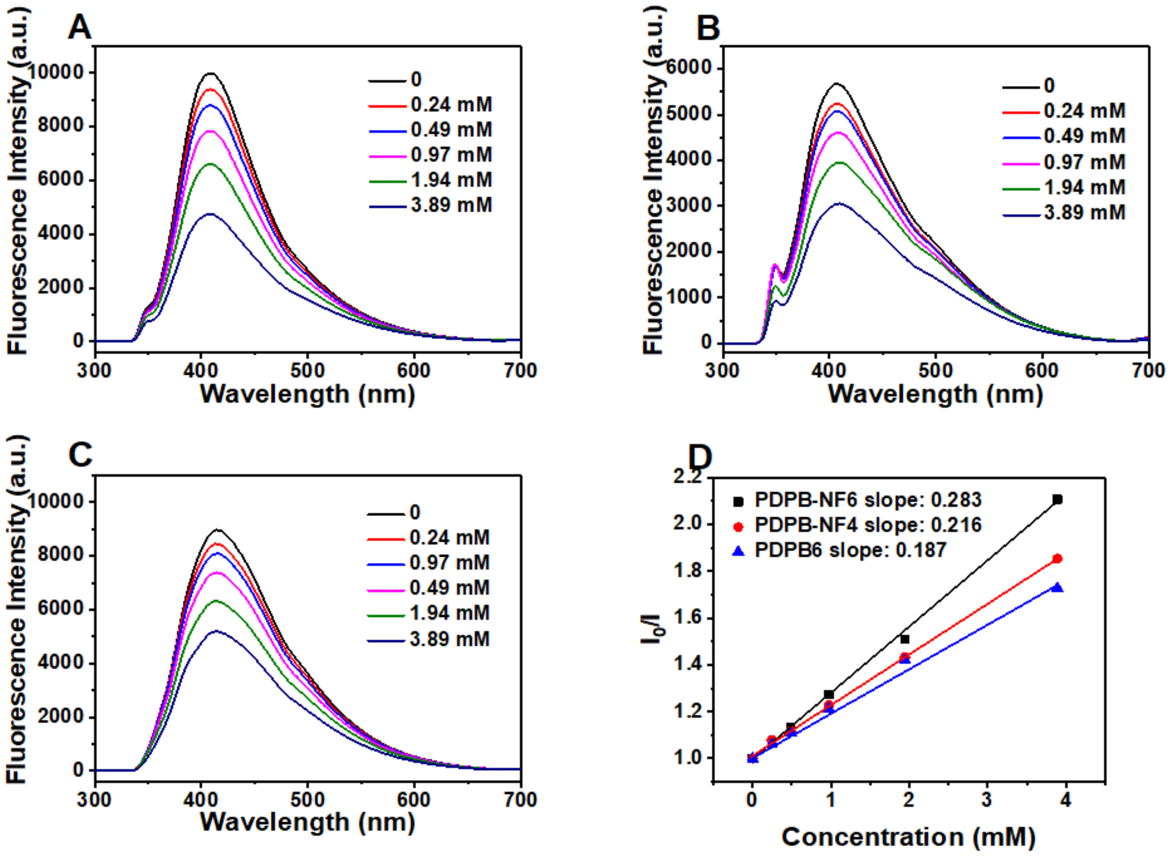

Figure S14. Fluorescence quenching study of (A) PDPB-NF6 and (B) PDPB-NF4 (C) PDPB6 in EtOH with varying concentrations of quencher, $MVCl_2$; D) plots of the ratio I_0/I versus quencher concentration. I_0 and I correspond to the emission intensity in the absence and presence of quencher, respectively.

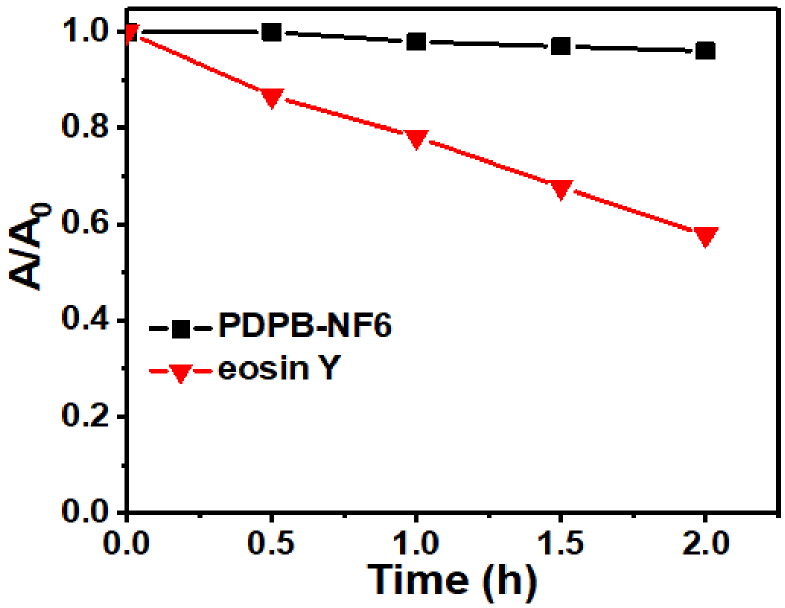

Figure S15. Photobleaching studies of eosin Y and PDPB-NF6 in DMF.

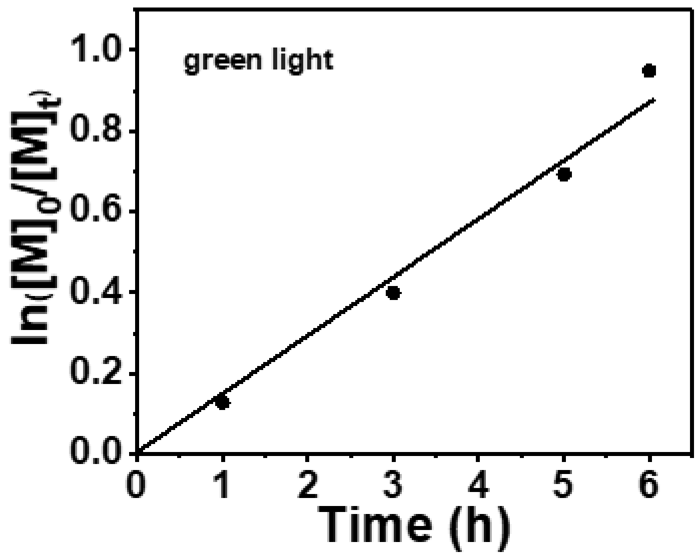

Figure S16. The plot of $\ln([M]_0/[M]_t)$ as a function of polymerization time, polymerization was conducted under green light irradiation. The apparent propagation rate $k_{p(\text{green light})} = 0.158$ h^{-1}.

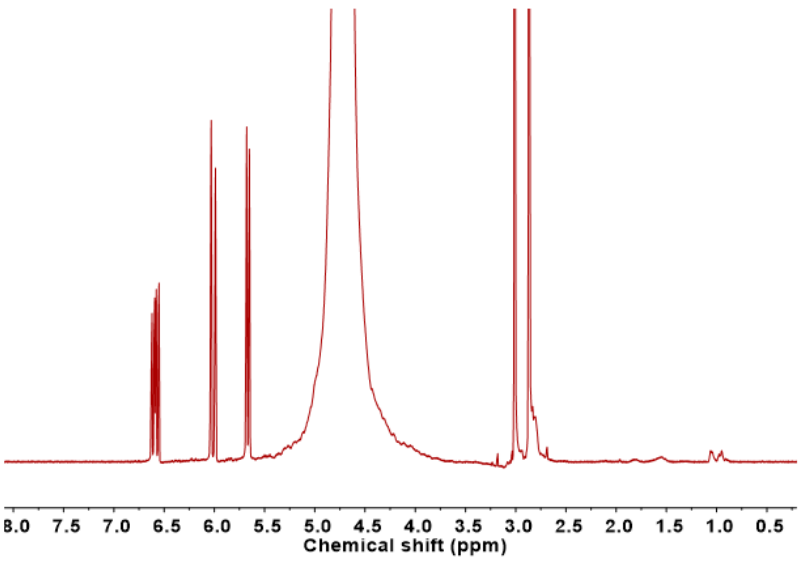

Figure S17. The ^{1}H-NMR spectrum of mixture after polymerization (Table 1, Entry 1).

Table S1. Examples of PDMA synthesized by photoinduced living polymerization catalysed by different photocatalysts.

Entry[a]	photocatalyst	$m_{photocatalyst}$ (mg)	concentration (mg/mL)	Conv[b] (%)	$M_{n.theo}$[b] (g/mol)	$M_{n.GPC}$ ($Đ$)[c] (g/mol)
1	PDPB-NF4	2.0	1.0	44.0	4638	2300 (1.25)
2	PDPB-NF4	1.4	0.7	42.9	4529	2010 (1.21)
3	PDPB-NF4	1.0	0.5	32.6	3509	1300 (1.18)
4	PDPB-NF6	2.0	1.0	61.2	6340	5210 (1.14)
5	PDPB-NF6	1.5	0.7	58.3	6053	5100 (1.17)
6	PDPB-NF6	1.0	0.5	54.1	5638	2330 (1.21)
7	PDPB6	2.0	1.0	52.3	5460	2470 (1.23)
8	PDPB6	1.4	0.7	44.1	4647	1740 (1.35)
9	PDPB6	1.0	0.5	39.5	4192	1500 (1.38)

[a] Reaction conditions: $[DMA]_0$:$[BDMAT]_0$ = 100:1, $[DMA]_0$ = 0.5 M, catalyst dispersed in 2 mL water, polymerization carried out at room temperature with visible light irradiation for 1.0 h. [b] Monomer conversion and $M_{n,theo}$ determined by ^{1}H NMR spectroscopy; $M_{n,theo} = [DMA]_0/[BDMAT]_0 \times MW_{DMA} \times Conv. + MW_{BDMAT}$, where $[DMA]_0$, $[BDMAT]_0$, MW_{DMA}, Conv., and MW_{BDMAT} represent the initial monomer concentration, initial BDMAT concentration, molar mass of DMA, monomer conversion, and molar mass of BDMAT. [c] $M_{n,GPC}$ and $Đ$ were determined by GPC with PS standards.

Table S2. Investigation of electron transfer mechanism using fast electron acceptor, methylviologen ($MVCl_2$).

Entry[a]	$[DMA]_0$:$[BADMAT]_0$:$[MVCl_2]_0$	Conv[b] (%)	$M_{n.theo}$[b] (g/mol)	$M_{n.GPC}$ ($Đ$)[c] (g/mol)
1	100:1	33.1	3599	1050 (1.28)
2	100:1:0.052	7.1	985	-[d]

[a] Reaction conditions: $[DMA]_0$:$[BDMAT]_0$ = 100:1, $[DMA]_0$ = 0.5 M, 1 mg PDPB-NF6 dispersed in 1 mL water, polymerization carried out at room temperature with visible light irradiation for 0.5 h. [b] Monomer conversion and $M_{n,theo}$ determined by ^{1}H NMR spectroscopy; $M_{n,theo} = [DMA]_0/[BDMAT]_0 \times MW_{DMA} \times$ Conv. + MW_{BDMAT}, where $[DMA]_0$, $[BDMAT]_0$, MW_{DMA}, Conv., and MW_{BDMAT} represent the initial monomer concentration, initial BDMAT concentration, molar mass of DMA, monomer conversion, and molar mass of BDMAT. [c] $M_{n,GPC}$ and $Đ$ were determined by GPC with PS standards. [d] Not determined.
